# The Quality of Life after Endometrial Cancer Study: Baseline Characteristics and Patient-Reported Outcomes

**DOI:** 10.3390/curroncol31090412

**Published:** 2024-09-17

**Authors:** Simrit Warring, Kathleen J. Yost, Andrea L. Cheville, Sean C. Dowdy, Stephanie S. Faubion, Amanika Kumar, Maureen A. Lemens, Chelsie C. Van Oort, Angela J. Fought, Michaela E. Mc Gree, Andrea Mariani, Gretchen Glaser

**Affiliations:** 1 Division of Gynecologic Oncology, Mayo Clinic, Rochester, MN 55902, USA; 2 Department of Quantitative Health Sciences, Mayo Clinic, Rochester, MN 55902, USA; 3 Department of Physical Medicine and Rehabilitation, Mayo Clinic, Rochester, MN 55902, USA; 4 Women’s Health Research Center, Mayo Clinic, Rochester, MN 55902, USA; 5 Department of Internal Medicine, Mayo Clinic, Jacksonville, FL 32224, USA; 6 Surgery Research, Division of Gynecology, Department of Obstetrics and Gynecology, Mayo Clinic, Rochester, MN 55902, USA; 7 Division of Clinical Trials and Biostatistics, Mayo Clinic, Rochester, MN 55902, USA

**Keywords:** quality of life, endometrial cancer, patient-reported outcomes, lower extremity lymphedema, sexual function and satisfaction, gynecologic oncology

## Abstract

Endometrial cancer (EC) patients make up the second largest group of female cancer survivors. Patient-reported outcomes (PROs) including quality of life (QOL) and sexual function and satisfaction (SF and S) are critical facets of survivorship. This prospective, longitudinal study assesses associations between baseline characteristics and PROs after treatment. Herein, we report the baseline clinical characteristics and PROs prior to treatment initiation. Outcomes post-treatment over time will be reported separately. Patients with planned surgery for EC were prospectively enrolled in 2019–2021 and administered the European Organization for Research and Treatment of Cancer (EORTC) QOL Questionnaire Core 30 (QLQ-C30), EORTC QLQ EC Module (EN24), Patient-Reported Outcomes Measurement Information System (PROMIS), and the Mayo Clinic lower extremity lymphedema (LEL) questionnaire. This study enrolled 198 patients with a mean (SD) age of 63.6 (9.8) years and body mass index of 35.5 (8.3) kg/m^2^. No significant differences in the PROs for the QOL were seen when compared to the reference means (SD) except for the lower interest in sexual activity (31.9 (9.8) vs. 47.5 (SE0.70)) and lower fatigue (21.3 (19.8) vs. 31.7 (25.9)). Increased obesity was associated with an increased likelihood of LEL (*p* < 0.01) and multiple QOL scales, including poorer global health status (*p* < 0.01) and physical functioning (*p* < 0.01). Prior to treatment initiation for EC, the patients had a similar QOL compared to that of the general population. The patients with increasing obesity, a known risk factor for EC, had worse overall global health status and physical functioning. A better understanding of these QOL measures is imperative for earlier identification and intervention of patients at risk of chronic impairments from EC treatment.

## 1. Introduction

Endometrial cancer (EC) is the most common gynecologic malignancy, with an estimated 66,200 new cases in the United States in 2023 [1]. Most patients with EC are diagnosed at a low stage and grade, leading to favorable long-term survival. As a result, this group makes up the second largest group of female cancer survivors [2,3]. Given the large percentage of patients with long survivorship periods, improving the quality of life (QOL) in patients with EC is of the utmost importance. The oncologic outcomes of EC have been well studied, but investigations into the impact on QOL and other measures of physical functioning have been lacking, especially in a prospective setting. The side effects of EC treatment can have long-term consequences and lead to increased unemployment and healthcare utilization [4,5]. Some of these side effects include lower extremity lymphedema (LEL), sexual dysfunction, and peripheral neuropathy [6,7,8]. In addition to therapy-related side effects, the common comorbidity of obesity has been shown to further negatively impact the QOL of patients with EC [9,10].

Patient-reported outcomes (PROs) inform both patients and healthcare providers of issues which can then be addressed and improved but have been inconsistently used in cancer survivors. To address this, the Patient-Reported Outcomes Measurement Information System (PROMIS^®^) has been developed by the National Institutes of Health (NIH) for use in patients with chronic conditions, a clear indicator that more of this type of research is needed [11]. The combination of these PROs with sociodemographic and clinical data could identify patients for whom chronic impairments from their EC treatment will negatively impact their lives and direct them to corrective therapy earlier than is currently possible. In addition, care management tools specifically geared towards patients with obesity could help inform providers regarding unique health issues in this complex cohort of patients with EC who are most at risk for diminished QOL.

The prospective Quality of Life after Endometrial Cancer (QLEC) study was developed to (i) describe QOL, sexual dysfunction, side effects of treatment, and LEL after lymph node assessment; (ii) assess the associations between baseline clinical data and subsequent QOL outcomes measured using PROs prior to treatment initiation and 6 months, 12 months, and 24 months after surgery. In this report, we describe the baseline pre-treatment patient characteristics and PROs of participants enrolled in the QLEC study prior to treatment initiation, including LEL, sexual function and satisfaction, and other functional and symptom-related QOL scales.

## 2. Methods

### 2.1. Study Design Overview

The QLEC study was approved by the Mayo Clinic Institutional Review Board (#18-011345). We performed a single institution prospective study of patients with planned surgery for newly diagnosed EC who underwent hysterectomy and sentinel lymph node (SLN) biopsy by any surgical modality at Mayo Clinic Rochester between 12 March 2019 and 28 January 2021. Patients agreed to collection of clinical data and QOL outcomes measured prior to surgery and at 6 months, 12 months, and 24 months after surgery. Patients who were ≥18 years old and English-speaking were included. Patients who received neoadjuvant therapy or had synchronous cancer at the time of diagnosis were excluded. Planned surgery included hysterectomy (laparotomy, laparoscopic, robotic, or vaginal) and lymph node assessment with SLN biopsy (using NCCN protocol [12]). All patients were administered the European Organization for Research and Treatment of Cancer (EORTC) Quality of Life Questionnaire Core 30 (QLQ-C30) [13] and EORTC QLQ Endometrial Cancer Module (EN24) [14] to assess QOL. Patient-Reported Outcomes Measurement Information System (PROMIS) assessed sexual function and satisfaction (SF and S) [15]. The 13-item Mayo Clinic LEL screening questionnaire [16] was used to assess LEL.

### 2.2. Baseline Measures

Baseline questionnaires (EORTC QLQ-C30, EORTC QLQ-EN24, PROMIS SF&S, and Mayo Clinic LEL) were administered in paper booklet form at the preoperative visit. All survey responses were entered into a Research Electronic Data Capture (REDCap) database. Patient demographics and clinical variables were manually abstracted from the electronic medical record (EMR).

### 2.3. Statistical Analysis

Prospectively abstracted data were summarized using standard descriptive statistics including frequencies and percentages for categorical variables and mean and standard deviation (SD) or median and interquartile range (IQR) for continuous variables. To assess generalizability of the study, patients enrolled versus those who declined enrollment were compared (see description in Supplemental Table S1). Each of the validated questionnaires was scored using published rules [13,14,16]. The QOL subscales from preoperative surveys were descriptively summarized. The prevalence of patients with preoperative LEL was reported and defined as a prorated score > 8 [17]. A poor outcome or score for the EORTC QLQ-C30 was defined as clinically meaningful for scores that were 10 points below the published norm for each scale/domain [13]. For PROMIS scales, 5 points on a T-score scale is considered a clinically meaningful difference [15]. We assessed the relationship between three BMI categories (BMI < 30, 30.0–39.9, and 40.0+ kg/m^2^) and screening positive for LEL, as well as survey responses for LEL questionnaire, PROMIS SF&S, EORTC QLQ-30, and EORTC QLQ-EN24.

## 3. Results

**Figure 1:**
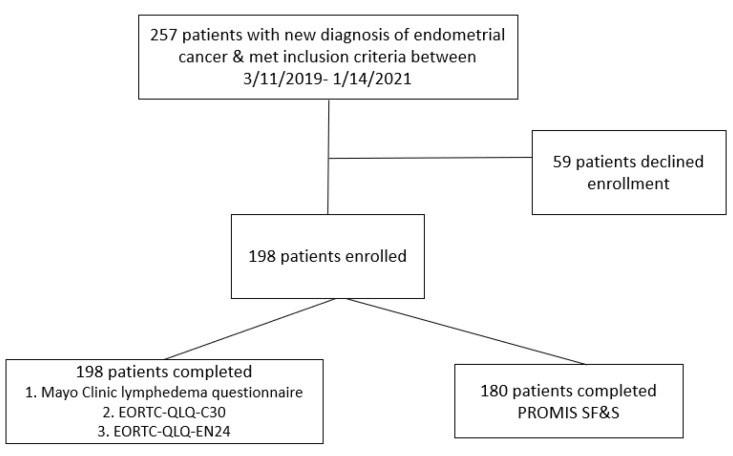
Eligibility and participant flow diagram.

During the study period, 257 patients had a planned surgery for newly diagnosed EC and met the inclusion criteria of the study. There were 198 patients within this cohort that were enrolled in the study and 59 patients that declined enrollment (Figure 1). The patient demographic characteristics in the enrolled and not enrolled groups are presented in Supplemental Table S1. The treatment characteristics of the enrolled patients are presented in Table 1. Most of the patients had robot-assisted surgery (n = 162, 81.8%) and International Federation of Gynecology and Obstetrics (FIGO 2009) stage IA disease (n = 154, 77.8%). Most of the surgeries included an SLN biopsy ± pelvic and/or paraaortic lymphadenectomy if the SLN failed to map (n = 186, 93.9%). The most common histology among the study patients was endometrioid (n = 179, 90.4%) and grade 1 (n = 120, 60.6%). Within 30 days of surgery, there were no reoperations and only two readmissions (1.0%).

A summary of baseline LEL questionnaire responses by BMI is presented in Table 2. The prevalence of screen-positive LEL (defined as a prorated score > 8) in the total study population was 13.1% (n = 26). A higher BMI was associated with an increased likelihood of LEL at baseline (*p* < 0.01). The prevalence of screen-positive LEL patients with BMI < 30 kg/m^2^, BMI 30–39.9 kg/m^2^, BMI 40+ kg/m^2^ was 1.8% (n = 1), 15.5% (n = 13), and 20.3% (n = 12), respectively.

A summary of the baseline PROMIS sexual function and satisfaction responses by BMI are presented in Table 3. A lower interest in sexual activity was seen in our patients at baseline when compared to the reference population (T-score mean: 31.9 (SD 9.8) vs. 47.5 (SE 0.70)). No meaningful differences were seen in the following domains: orgasm ability, orgasm pleasure, satisfaction with sex life, and vaginal lubrication/vaginal discomfort/labial discomfort/clitoral discomfort with sexual activity.

Table 4 provides a summary of the functional and symptom QOL scales from EORTC QLQ-C30 by BMI. There were no significant differences (defined as a 10-point difference from the reference mean) seen between the study population and the reference mean (SD) for the functional or symptom scales except for fatigue (21.3 (19.8) vs. 31.7 (25.9)). An association between a poorer global health status (*p* < 0.01) and poorer physical functioning (*p* < 0.01) with increasing BMI was observed. A relationship between higher BMI and worse fatigue (*p* = 0.02) and dyspnea (*p* < 0.01) was also observed.

A summary of the functional and symptom QOL scales from EORTC QLQ-EN24 by BMI is presented in Table 5. There was a significant association between better sexual interest functioning and lower BMI. A relationship between higher BMI and worse symptoms was seen in the following symptom scales: lymphedema (*p* < 0.01), urological (<0.01), pain in back and pelvis (*p* = 0.02), tingling/numbness (*p* < 0.01), and muscular pain (*p* = 0.01).

## 4. Discussion

There are limited studies available that report baseline QOL PROs in patients with endometrial cancer prior to treatment. In our study population, we observed less sexual interest and fatigue than the general population, but otherwise a similar QOL in comparison to reference groups for EORTC QLQ-C30 and PROMIS SF&S. The results from our study also show that patients with EC and increasing obesity are found to have worse overall global health status and physical functioning, as well as significantly increased LEL, fatigue, dyspnea, urological symptoms, back/pelvic pain, tingling/numbness, and general muscular pain.

Cancer-related fatigue represents one of the most prevalent effects of cancer and the treatment of cancer can further reduce the overall QOL [18,19]. Fatigue has been identified in patients with cancer at all stages, including diagnosis and treatment. For example, in a longitudinal study of breast cancer patients followed before surgery to 8 months after surgery, the mean fatigue level prior to surgery was higher than that in the general US population [20]. In our study, we found that patients with EC had less fatigue prior to treatment initiation when compared to the general population. One speculation for this unexpected finding may be that patients with early EC do not typically have the same systemic symptoms associated with other cancers, and thus may feel similar to their baseline. It will be elucidative to assess how this QOL measure changes post-treatment with our longitudinal data.

Sexual function is a multifaceted outcome, with several contributing factors, both physical and psychological. Studies examining sexual function after gynecologic cancer diagnoses have largely focused on symptoms during or after treatment, and have not focused solely on patients with EC. In these patients, post-treatment sexual dysfunction is reported to be higher than in patients without gynecologic cancer [21,22,23,24,25,26,27,28]. In an EC-specific study, 55.9% of the patients never returned to being sexually active after surgery [21,22,29,30]. The findings from our study suggest that sexual dysfunction, specifically a lack of interest in sexual activity, may be present prior to treatment initiation and therefore not entirely related to post-treatment-specific side effects alone. This knowledge could help cancer care teams address and normalize decreased sexual interest even before EC treatment begins.

Obesity, one of the known risk factors for low-grade endometrial cancer, has been shown in prior studies to negatively affect sexual function. One cross-sectional study analyzing records of cisgender women seen for specialty consultation in women’s health clinics showed that being overweight or obese was associated with a lack of sexual activity [31]. Among sexually active women, overweight or obese women had worse sexual function based on lower Female Sexual Function Index scores and sexual function domain scores compared to women of normal weight. This included worse sexual arousal, lubrication, satisfaction, orgasm, pain, and sexual distress. A multivariable analysis, however, revealed the associations were indirect and mediated by other factors known to impact body weight and sexual function, including age, level of education, reproductive stage, medication use, and mood disturbance. Our study did not demonstrate this in the baseline data, and this will be closely assessed in our longitudinal data.

Another QOL-related symptom affected by obesity is LEL. We observed an increased likelihood of LEL in patients with obesity that were recently diagnosed with EC but had not yet initiated treatment. This was found using a prorated score > 8, thought to be more accurate than the original cut-off point of 4, especially in obese patients. The Mayo Clinic LEL screening questionnaire was initially validated with a score threshold of >4 points for identifying LEL [8]. A subsequent study suggested a higher threshold of >8 may be warranted [17], especially among obese women, since obesity is an independent risk factor for general edema due to cardiac or renal impairment as well as chronic venous insufficiency, lymphatic abnormalities, and generalized inflammation [32,33]. LEL is a well-known sequela of EC treatment, either related to lymph node dissection or pelvic radiation, though our results suggest that the risk of pre-existing self-reported LEL unrelated to treatment sequela is increased in patients with obesity. This poses the concern that patients with obesity could be at additional risk of EC treatment further worsening pre-existing LEL and its associated morbidity, which could further impair physical functioning during their survivorship. Furthermore, this finding in our baseline data emphasizes the importance of adjusting for pre-treatment LEL screening scores when assessing incident LEL in the prospective data.

Patients in our study with increasing classes of obesity also reported lower overall global health status and physical functioning scores, as well as more fatigue and dyspnea on symptoms scales. When evaluating EC survivorship, previous studies have shown that an elevated BMI has been associated with a decreased QOL compared to normal weight controls [8,34,35,36,37,38,39,40]. Evidence supports that a decreased QOL could be related to lower activity levels in this group of patients [36]. Evidence from a systematic review of obesity and EC survivors shows statistically significant associations between obesity and all-cause mortality [41]. Consistent with findings from our study, it has previously been reported that there is a higher prevalence of musculoskeletal pain in patients with obesity and an increased BMI is an independent risk factor for musculoskeletal pain disorders [42,43]. Also consistent with our study findings is that multiple studies have reported on the relationship between an increased prevalence of peripheral neuropathy in obese patients, even those with normoglycemia [44,45]. Neuropathy can lead to increased pain, falls, and a lower quality of life [46]. Each of the symptoms that our study found to be associated with higher BMI in patients recently diagnosed with EC can negatively impact an individual patient’s post-treatment recovery, therefore putting these EC survivors at a higher risk for further decreased QOL.

The strengths of our study include the use of multiple validated questionnaires to address various aspects of QOL and physical functioning. The large sample size allows for clinically meaningful differences to be identified. The prospective nature of the study design limits recall bias. The limitations of the study include the lack of racial diversity in the overall patient cohort approached for study enrollment (98.8% white) and that this was a single institution study.

In conclusion, patients recently diagnosed with EC who have not yet initiated treatment have a similar QOL in both functional and symptom domains compared to the general population with the exception of having lower interest in sexual activity and less fatigue. A statistically significant association was observed between increasing classes of obesity and conditions known to affect the QOL, such as a positive screen for LEL and having poorer overall global health status, physical functioning, and sexual interest. An increased likelihood of fatigue, dyspnea, lymphedema, urological symptoms, back and pelvic pain, tingling/numbness, and muscular pain-related symptoms were also seen in the pre-treatment EC patients with increasing obesity. With this knowledge, healthy behavioral and lifestyle counseling to promote weight loss should be the standard of care in patients with endometrial cancer and obesity.

Understanding the baseline QOL in patients with EC prior to treatment initiation will help to inform the development of a risk assessment algorithm that identifies patients at the highest risk of poor QOL outcomes. We anticipate that in the future, this risk assessment algorithm can be automated into the EMR and serve to notify care teams of which patients are at the highest risk of adverse health outcomes. These investigations will then inform development of a centralized, collaborative telecare model for screening for, education in, and treatment of adverse health outcomes in patients being treated for EC.

## Figures and Tables

**Table 1:** Baseline demographics and surgical and treatment characteristics of enrolled patients.

Demographic Characteristics	n = 198
Age (years), mean (SD)	63.6 (9.8)
BMI (kg/m^2^), median (IQR)	36.0 (29.3, 41.5)
Race	
White	197 (99.5)
Other	1 (0.5)
Education	
High school graduate/GED or less	44 (22.2)
Some college or 2-year degree	71 (35.9)
4-year degree	54 (27.3)
Master’s or Ph.D.	27 (13.6)
Unknown	2 (1.0)
ASA score	
≤2	101 (51.0)
>2	97 (49.0)
**Treatment characteristics**	
**Procedure type**	
Laparotomy	14 (7.1)
Vaginal	6 (3.0)
Robot-assisted	162 (81.8)
Laparoscopic	16 (8.1)
**Procedures**	
Oophorectomy	191 (96.5)
Pelvic LND	21 (10.6)
Paraaortic LND	4 (2.0)
SLN biopsy ± LND	186 (93.9)
Number of pelvic nodes removed (n = 21), mean (SD)	11.5 (7.6)
Number of paraaortic nodes removed (n = 4), mean (SD)	8.3 (5.6)
SLN nodes removed (n = 186), mean (SD)	3.3 (2.2)
**Disease characteristics**	
**Histology**	
Endometrioid	179 (90.4)
Serous	7 (3.5)
Clear cell	2 (1.0)
No residual cancer identified	4 (2.0)
Other *	6 (3.0)
**Stage (FIGO 2009)**	
IA	154 (77.8)
IB	20 (10.1)
II	6 (3.0)
IIIA	6 (3.0)
IIIC	2 (1.0)
IVA	1 (0.5)
No residual cancer identified	9 (4.5)
**Grade**	
Grade 1	120 (60.6)
Grade 2	53 (26.8)
Grade 3	8 (4.0)
Not applicable	17 (8.6)
**Postoperative outcomes**	
Readmission within 30 days of surgery	2 (1.0)
Reoperation within 30 days of surgery	0 (0.0)
**Adjuvant therapy**	
None	149 (75.3)
VB only	27 (13.6)
EBRT ± VB	6 (3.0)
Chemotherapy ± VB	5 (2.5)
Chemotherapy and EBRT ± VB	11 (5.6)

Abbreviations: ASA, American Society of Anesthesiologists; BMI, body mass index, GED, general educational development; EBRT, external beam radiation therapy; FIGO, International Federation of Gynecology and Obstetrics; IQR, interquartile range; LND, lymphadenectomy; SD, standard deviation; SLN, sentinel lymph node; VB, vaginal brachytherapy. Results presented as N (%) unless otherwise stated. * Other includes complex atypical hyperplasia (N = 3), mesonephric carcinoma (N = 1), adenocarcinoma in situ notified (N = 1), and scan benign atrophic endometrium, benign cervix, ovaries, and fallopian tubes (N = 1).

**Table 2:** Summary of baseline lymphedema questionnaire by body mass index.

Characteristic	Total (N = 198)	Underweight/ Normal/Overweight (BMI < 30.0 kg/m^2^) (N = 55)	Obesity Class I/II (BMI 30.0–39.9 kg/m^2^) (N = 84)	Obesity Class III (BMI 40.0+ kg/m^2^) (N = 59)	*p* *
Total # participants completed survey	198	55	84	59	
LEL screen-positive (prorated score > 4), N (%)	63 (31.8)	8 (14.5)	28 (33.3)	27 (45.8)	<0.01
LEL screen-positive (prorated score > 8), N (%)	26 (13.1)	1 (1.8)	13 (15.5)	12 (20.3)	<0.01

Abbreviations: BMI, body mass index; LEL, lower extremity lymphedema. Results presented based on non-missing values. * Comparisons across body mass index groups utilized the chi-square or Fisher’s exact test *p* value.

**Table 3:** Baseline PROMIS sexual function and satisfaction by body mass index.

Domain *	Total		T-Score, Mean (SE) ^†(1)^	Underweight/ Normal/Overweight (BMI < 30.0 kg/m^2^) (N = 51)	Obesity Class I/II (BMI 30.0–39.9 kg/m^2^) (N = 77)	Obesity Class III (BMI 40.0+ kg/m^2^) (N = 52)	*p* ^‡^
	N	T-Score, Mean (SD)		T-Score, Median (IQR)	T-Score, Median (IQR)	T-Score, Median (IQR)	
Interest in sexual activity	180	31.9 (9.8)	47.5 (0.70)	32.9 (21.9, 43.9)	27.4 (21.9, 38.4)	32.9 (21.9, 38.4)	0.26
Orgasm ability	42	43.6 (11.2)	47.4 (0.74)	39.6 (30.2, 58.4)	39.6 (39.6, 49.0)	49.0 (39.6, 53.7)	0.48
Orgasm pleasure	39	44.6 (7.1)	48.8 (0.84)	47.5 (40.2, 58.6)	40.2 (40.2, 47.5)	43.9 (40.2, 47.5)	0.61
Satisfaction with sex life	46	46.8 (6.8)	47.5 (0.86)	45.3 (39.8, 51.9)	45.3 (39.8, 51.9)	45.3 (42.5, 51.9)	0.93
Vaginal lubrication for sexual activity	43	49.7 (8.7)	46.2 (0.83)	51.2 (40.3, 51.2)	50.2 (41.8, 58.4)	58.4 (51.9, 58.4)	0.11
Vaginal discomfort with sexual activity	42	50.5 (8.6)	51.9 (0.80)	53.3 (43.3, 57.1)	48.1 (43.3, 56.9)	43.3 (43.3, 48.3)	0.27
Labial discomfort with sexual activity	44	50.4 (6.5)	50.9 (0.92)	47.4 (47.4, 47.4)	47.4 (47.4, 47.4)	47.4 (47.4, 47.4)	0.81
Clitoral discomfort with sexual activity	44	50.2 (5.8)	50.4 (0.81)	48.2 (48.2, 48.2)	48.2 (48.2, 48.2)	48.2 (48.2, 48.2)	0.99

Abbreviations: BMI, body mass index; IQR, interquartile range; SD, standard deviation; SE, standard error. * Higher scores in interest in sexual activity indicate greater interest, higher scores in orgasm ability indicated greater ability to have an orgasm, higher scores in orgasm pleasure indicate more pleasurable orgasms, higher scores in satisfaction with sex life indicate more satisfying sexual activities, higher scores in vaginal lubrication for sexual activity indicate greater lubrication, and higher scores in discomfort domains indicate greater discomfort. ^†(1)^ Table 5 for age 60 or older for sexually active U.S. adults. ^‡^ Comparisons between the body mass index groups were evaluated using the Kruskal–Wallis Test.

**Table 4:** Summary of functional and symptom QOL scales from EORTC QLQ-C30 by body mass index.

	Total (N = 198)	Reference Mean (SD) ^(2)^	Underweight/Normal/ Overweight (BMI < 30.0 kg/m^2^) (N = 55)	Obesity Class I/II (BMI 30.0–39.9 kg/m^2^) (N = 84)	Obesity Class III (BMI 40.0+ kg/m^2^) (N = 59)	*p* *
**Functional scales ^†^**						
**Global health status**						<0.01
Mean (SD)	72.6 (17.6)	64.3 (21.8)	79.5 (15.9)	74.6 (16.6)	63.1 (16.8)	
Median (IQR)	75.0 (66.7, 83.3)		83.3 (66.7, 91.7)	75.0 (66.7, 83.3)	66.7 (58.3, 75.0)	
No. score of 100, N (%)	13 (6.6)		7 (12.7)	6 (7.1)	3 (5.1)	
**Physical functioning**						<0.01
Mean (SD)	87.5 (16.5)	84.3 (18.5)	94.7 (8.5)	87.8 (15.5)	80.3 (20.2)	
Median (IQR)	93.3 (86.7, 100.0)		100.0 (93.3, 100.0)	93.3 (86.7, 100.0)	86.7 (73.3, 93.3)	
No. score of 100, N (%)	84 (42.4)		34 (61.8)	36 (42.9)	14 (23.7)	
**Role functioning**						0.08
Mean (SD)	91.9 (17.6)	84.1 (24.6)	95.5 (12.2)	92.3 (18.0)	87.9 (20.7)	
Median (IQR)	100.0 (100.0, 100.0)		100.0 (100.0, 100.0)	100.0 (100.0, 100.0)	100.0 (83.3, 100.0)	
No. score of 100, N (%)	152/197 (77.2)		47 (85.5)	66 (78.6)	39/58 (67.2)	
**Emotional functioning**						0.46
Mean (SD)	76.3 (19.8)	71.9 (25.3)	75.3 (18.5)	77.6 (20.5)	75.3 (20.3)	
Median (IQR)	83.3 (66.7, 91.7)		83.3 (66.7, 91.7)	83.3 (66.7, 91.7)	83.3 (66.7, 91.7)	
No. score of 100, N (%)	27/196 (13.8)		6/54 (11.1)	16/83 (19.3)	5 (8.5)	
**Cognitive functioning**						0.42
Mean (SD)	86.5 (18.7)	84.3 (20.9)	89.2 (14.5)	86.7 (17.8)	83.6 (22.8)	
Median (IQR)	100.0 (83.3, 100.0)		100.0 (83.3, 100.0)	100.0 (83.3, 100.0)	83.3 (83.3, 100.0)	
No. score of 100, N (%)	101/196 (51.5)		29/54 (53.7)	44/83 (53.0)	28 (47.5)	
**Social functioning**						0.57
Mean (SD)	87.2 (21.8)	85.7 (24.6)	89.8 (16.3)	89.6 (19.1)	81.4 (28.2)	
Median (IQR)	100.0 (83.3, 100.0)		100.0 (83.3, 100.0)	100.0 (83.3, 100.0)	100.0 (66.7, 100.0)	
No. score of 100, N (%)	126/196 (64.3)		36/54 (66.7)	58/83 (69.9)	32 (54.2)	
**Symptom scales and items ^‡^**						
**Fatigue**						0.02
Mean (SD)	21.3 (19.8)	31.7 (25.9)	15.2 (14.2)	22.4 (21.1)	25.4 (21.3)	
Median (IQR)	22.2 (11.1, 33.3)		11.1 (0.0, 22.2)	22.2 (11.1, 33.3)	22.2 (11.1, 33.3)	
No. score of 0, N (%)	47 (23.7)		21 (38.2)	20 (23.8)	6 (10.2)	
**Pain**						0.06
Mean (SD)	17.9 (23.7)	25.3 (27.9)	13.6 (22.2)	18.7 (24.0)	20.9 (24.5)	
Median (IQR)	16.7 (0.0, 33.3)		0.0 (0.0, 16.7)	16.7 (0.0, 33.3)	16.7 (0.0, 33.3)	
No. score of 0, N (%)	97 (49.0)		32 (58.2)	39 (46.4)	26 (44.1)	
**Dyspnea, N (%)**						<0.01
0	149 (75.3)		50 (90.9)	66 (78.6)	33 (55.9)	
33.3	45 (22.7)		5 (9.1)	17 (20.2)	23 (39.0)	
66.7	3 (1.5)		-	1 (1.2)	2 (3.4)	
100	1 (0.5)		-	-	1 (1.7)	

Abbreviations: BMI, body mass index; IQR, interquartile range; QOL, quality of life; SD, standard deviation. * Comparisons between the BMI groups were evaluated using the Kruskal–Wallis test. ^†^ Measured on a 0–100 point scale; higher functional scores indicate better functional well-being. ^‡^ Measured on a 0–100 point scale; higher symptom scores indicate worse symptom.

**Table 5:** Summary of functional and symptom QOL scales from EORTC QLQ-EN24 by body mass index.

Scale	Total (N = 198)	Underweight/ Normal/ Overweight (BMI < 30.0 kg/m^2^) (N = 55)	Obesity Class I/II (BMI 30.0–39.9 kg/m^2^) (N = 84)	Obesity Class III (BMI 40.0+ kg/m^2^) (N = 59)	*p* *
**Functional scales ^†^**					
**Sexual interest functioning**					0.02
Mean (SD)	18.5 (21.5)	25.6 (23.4)	15.4 (21.7)	16.4 (18.0)	
Median (IQR)	0.0 (0.0, 33.3)	33.3 (0.0, 33.3)	0.0 (0.0, 33.3)	0.0 (0.0, 33.3)	
No. score of 100, N (%)	1/191 (0.5)	1/52 (1.9)	0/82 (0.0)	0/57 (0.0)	
**Sexual activity functioning**					0.19
Mean (SD)	10.8 (19.0)	15.0 (23.4)	10.6 (18.1)	7.5 (15.3)	
Median (IQR)	0.0 (0.0, 33.3)	0.0 (0.0, 33.3)	0.0 (0.0, 33.3)	0.0 (0.0, 0.0)	
No. score of 100, N (%)	1/191 (0.5)	1/51 (2.0)	0/82 (0.0)	0/58 (0.0)	
**Sexual enjoyment functioning** **^¥^**					0.40
Mean (SD)	57.6 (26.4)	56.9 (32.8)	54.0 (24.7)	66.7 (15.7)	
Median (IQR)	66.7 (33.3, 66.7)	66.7 (33.3, 66.7)	66.7 (33.3, 66.7)	66.7 (66.7, 66.7)	
No. score of 100, N (%)	7/48 (14.6)	4/17 (23.5)	2/21 (9.5)	1/10 (10.0)	
**Symptom scales ^‡^**					
**Lymphedema**					<0.01
Mean (SD)	9.1 (15.0)	2.1 (7.2)	10.3 (15.5)	13.8 (17.3)	
Median (IQR)	0.0 (0.0, 16.7)	0.0 (0.0, 0.0)	0.0 (0.0, 16.7)	0.0 (0.0, 33.3)	
No. score of 0, N (%)	133 (67.2)	50 (90.9)	53 (63.1)	30 (50.8)	
**Urological**					<0.01
Mean (SD)	19.3 (19.6)	13.8 (15.7)	18.4 (19.4)	25.8 (21.6)	
Median (IQR)	16.7 (0.0, 25.0)	8.3 (0.0, 25.0)	16.7 (0.0, 25.0)	25.0 (8.3, 41.7)	
No. score of 0, N (%)	52 (26.3)	18 (32.7)	24 (28.6)	10 (16.9)	
**Gastrointestinal**					0.17
Mean (SD)	13.8 (14.0)	11.3 (13.1)	15.4 (15.0)	13.9 (13.2)	
Median (IQR)	13.3 (0.0, 20.0)	6.7 (0.0, 13.3)	13.3 (6.7, 20.0)	13.3 (6.7, 20.0)	
No. score of 0, N (%)	51 (25.8)	18 (32.7)	19 (22.6)	14 (23.7)	
**Poor body image**					0.92
Mean (SD)	8.6 (16.6)	8.3 (17.1)	8.1 (14.3)	9.6 (19.4)	
Median (IQR)	0.0 (0.0, 16.7)	0.0 (0.0, 0.0)	0.0 (0.0, 16.7)	0.0 (0.0, 16.7)	
No. score of 0, N (%)	145/197 (73.6)	41/54 (75.9)	60 (71.4)	44 (74.6)	
**Sexual/vaginal problems** **^¥^**					0.25
Mean (SD)	17.1 (23.1)	26.1 (30.4)	13.8 (17.5)	8.9 (14.6)	
Median (IQR)	11.1 (0.0, 27.8)	22.2 (0.0, 33.3)	11.1 (0.0, 22.2)	0.0 (0.0, 22.2)	
No. score of 0, N (%)	23/48 (47.9)	7/17 (41.2)	9/21 (42.9)	7/10 (70.0)	
**Pain in back and pelvis**					0.02
Mean (SD)	29.5 (28.7)	20.0 (23.7)	34.9 (31.4)	30.5 (27.2)	
Median (IQR)	33.3 (0.0, 33.3)	0.0 (0.0, 33.3)	33.3 (0.0, 50.0)	33.3 (0.0, 66.7)	
No. score of 0, N (%)	76 (38.4)	29 (52.7)	26 (31.0)	21 (35.6)	
**Tingling/numbness**					<0.01
Mean (SD)	14.5 (22.9)	9.1 (19.7)	13.1 (21.3)	21.5 (26.1)	
Median (IQR)	0.0 (0.0, 33.3)	0.0 (0.0, 0.0)	0.0 (0.0, 33.3)	0.0 (0.0, 33.3)	
No. score of 0, N (%)	131 (66.2)	43 (78.2)	57 (67.9)	31 (52.5)	
**Muscular pain**					0.01
Mean (SD)	25.5 (26.1)	17.6 (23.0)	26.3 (25.7)	31.6 (28.0)	
Median (IQR)	33.3 (0.0, 33.3)	0.0 (0.0, 33.3)	33.3 (0.0, 33.3)	33.3 (0.0, 66.7)	
No. score of 0, N (%)	82/195 (42.1)	31 (56.4)	31/81 (38.3)	20 (33.9)	
**Hair loss**					0.10
Mean (SD)	9.6 (18.5)	5.5 (12.4)	12.3 (20.6)	9.8 (19.8)	
Median (IQR)	0.0 (0.0, 33.3)	0.0 (0.0, 0.0)	0.0 (0.0, 33.3)	0.0 (0.0, 0.0)	
No. score of 0, N (%)	147/197 (74.6)	46 (83.6)	57 (67.9)	44/58 (75.9)	
**Taste change**					0.40
Mean (SD)	4.0 (13.7)	1.8 (7.6)	5.6 (17.8)	4.0 (10.9)	
Median (IQR)	0.0 (0.0, 0.0)	0.0 (0.0, 0.0)	0.0 (0.0, 0.0)	0.0 (0.0, 0.0)	
No. score of 0, N (%)	178 (89.9)	52 (94.5)	74 (88.1)	52 (88.1)	

Abbreviations: BMI, body mass index; IQR, interquartile range; SD, standard deviation. * Comparisons between the BMI groups were evaluated using the Kruskal–Wallis test. ^†^ Measured on a 0–100 scale; higher functional scores indicate better functional well-being. ^‡^ Measured on a 0–100 scale; higher symptom scores indicate worse symptom. ^¥^ Only answered if patient indicated they had been sexually active during the past 4 weeks (a little, quite a bit, or very much).

## Data Availability

The raw data supporting the conclusions of this article will be made available by the authors on request.

## Supplementary Materials

The following supporting information can be downloaded at: https://www.mdpi.com/article/10.3390/curroncol31090412/s1, Table S1. Baseline demographic characteristics of study cohorts (enrolled vs. not enrolled) [47,48,49,50].
