# Supplementary material for: The Quality of Life after Endometrial Cancer Study: Baseline Characteristics and Patient-Reported Outcomes
Source: Curr Oncol. 2024 Sep 17;31(9):5557–72. doi: 10.3390/curroncol31090412 (PMC11431380; doi:10.3390/curroncol31090412)
Supplement: Supplementary file 1 [file curroncol-31-00412-s001.zip › curroncol-3192915 - supplementary materials.pdf]

Supplemental Table

Table S1. Baseline demographic characteristics of study cohorts (enrolled vs. not enrolled).

**Demographic Characteristics**	**Overall, n = 257**	**Enrolled, n = 198**	**Not enrolled, n = 59**	***p* ***
Age (years), mean (SD)	64.4 (9.8)	63.6 (9.8)	66.9 (9.3)	0.03
BMI (kg/m^2)				0.14
N	254	198	56	
Median (IQR)	35.3 (28.2, 41.3)	36.0 (29.3, 41.5)	31.3 (27.4, 41.0)	
Race				0.13
White	254 (98.8)	197 (99.5)	57 (96.6)	
Other	3 (1.2)	1 (0.5)	2 (3.4)	
Education				<0.01
High school graduate/GED or less	69 (26.8)	44 (22.2)	25 (42.4)	
Some college or 2-year degree	81 (31.5)	71 (35.9)	10 (16.9)	
4-year degree	58 (22.6)	54 (27.3)	4 (6.8)	
Master's or Ph.D.	31 (12.1)	27 (13.6)	4 (6.8)	
Unknown	18 (7.0)	2 (1.0)	16 (27.1)	
ASA score				0.16
≤2	125 (48.6)	101 (51.0)	24 (40.7)	
>2	132 (51.4)	97 (49.0)	35 (60.3)	

Abbreviations: ASA, American Society of Anesthesiologists; BMI, body mass index, GED, general educational development; IQR, interquartile range; SD, standard deviation. Results presented as N (%) unless otherwise stated. * Comparisons between enrolled and not enrolled utilized the t-test for age, Kruskal–Wallis test for BMI, and the chi-square or Fisher's exact test *p* value for categorical variables.

A comparison between the enrolled and not enrolled groups revealed clinically similar mean ages for the patients though these were statistically higher in the 'not enrolled' group (mean 63.6 vs. 66.9 years, $p = 0.03$). The 'not enrolled' patients had less education compared to the patients who were 'enrolled' (42.4% vs. 22.2% high school graduate/GED or less and 13.6% vs. 40.9% with 4-year degree, Master's degree, or PhD, respectively; $p < 0.01$). No differences were observed in the BMI, race, or ASA score. By comparing the baseline characteristics of the 'enrolled' and 'not enrolled' patients, we identified that the patients were clinically similar in age, with no differences in BMI, race, or ASA score, but the 'enrolled' patients did tend to have higher education levels. The trend towards higher education levels of the 'enrolled' patients is consistent with the United States Census Bureau in 2022, which uses the Annual Social and Economic Supplement of the Current Population Survey statistics. The highest level of education attainment by adults aged 25 and older in the US was a high school diploma or less in 37% and some college or higher in 62% [47]. Previous studies exploring the attributes of patient willingness to participate in clinical research have shown that the education level correlates with a patient's likelihood to participate in clinical studies, which is also consistent with our experience [48–50].
